# Intracellular acting tumor cell-targeted chemotherapy by MSC-suicide gene exosomes

**DOI:** 10.18632/oncotarget.27135

**Published:** 2019-09-24

**Authors:** Cestmir Altaner, Ursula Altanerova, Jana Jakubechova

**Affiliations:** Department of Molecular Oncology, Cancer Research Institute, Biomedical Center, Slovak Academy of Sciences, Bratislava, Slovakia; Stem Cell Preparation Department, St. Elisabeth Cancer Institute, Bratislava, Slovakia

**Keywords:** mesenchymal stem/stromal cells, exosome, suicide gene, yCD::UPRT, 5-fluorocytosine, 5-fluorouracil

Patients’ survival for most cancer types is slightly improving due to progress in faster diagnosis and advances in treatment. However, median period of survival time of patients with pancreatic cancer, glioblastoma, metastatic melanoma, metastatic lung tumors, high-grade serous subtype epithelial ovarian cancer, abdominal bowel and esophagus tumors treated with standard therapies is extremely low. There are several reasons for this therapeutic failure. Tumor initiating cells are resistant to drugs, have the ability of self-renewal and can be source of metastatic spread. Other additional major drawbacks of standard therapies lie in a lack of tumor specificity of anti-cancer drugs and the emergence of drug-resistant cell subpopulations after radio and/or chemotherapy. Further progress in cancer therapy (mainly aggressive tumors, metastases) requires novel therapeutic modalities.

Physiological role of human mesenchymal/stromal stem cells (MSCs) is to regenerate damaged and used tissues in the body. MSCs possess the ability to migrate to the site of injury and secrete a variety of soluble factors and extra-cellular nanovesicles – exosomes capable of a number of functions inducing and supporting regenerative processes in the damaged tissue. MSCs recognize tumor as an injury, home in the tumor and, together with other cells, form the tumor stroma. Based on our experience with virus mediated cancer gene therapy, the tumor-specific tropism of MSCs inspired us to develop a targeted prodrug gene therapy mediated by MSCs engineered to express the yeast cytosine deaminase::uracil phosphoribosyl transferase fusion gene (yCD::UPRT), yCD::UPRT-MSC/5-FC system [1] and MSCs expressing thymidine kinase of Herpes simplex virus with ganciclovir (GCV) as a prodrug – tk*HSV*-MSC/GCV system [2]. Both systems used retrovirus vector allowing its integration into cell DNA as a retroviral provirus and efficient expression of the suicide gene due to strong retrovirus promoter. In addition, the design of our retrovirus vector being a bicistronic construct with suicide gene separated by internal ribosome entry site sequence from neo gene, allows selecting homogenous population of transduced cells by the cell selection with G418 antibiotic [3]. Specifically, MSCs engineered to express yCD gene allows cells to convert nontoxic 5-fluorocytosine (5-FC) into cytotoxic 5-fluorouracil (5-FU) and the UPRT part of enzyme catalyses the direct conversion of 5-FU to 5-fluorouridinemonophosphate (5-FUMP) an irreversible inhibitor of DNA synthesis. The prodrug gene therapy mediated by MSCs has effectively inhibited the growth of human colon carcinoma [1] melanoma [4] and prostate carcinoma [5] in nude mice. AT-MSCs engineered to express yCD::UPRT gene were shown to induce curative therapeutic effect in substantial number of rats with intracranial glioblastoma in preclinical model [6]. In all these preclinical studies, we demonstrated that the therapeutic cells injected intravenously were effective in significantly inhibiting tumor growth in a dose dependent manner, despite the difficulty to find them at tumor site.

The explanation came when we found release of extracellular vesicles - exosomes from yCD::UPRT gene transduced MSCs. Exosomes incorporated mRNA of suicide gene in their cargo. Such exosomes we call MSC suicide gene exosomes. Size-exclusion chromatography of the secretome released from yCD::UPRT-MSCs revealed that the biological activity was clearly localized in the exosome fractions. These exosomes migrate to tumor similarly like MSCs, internalized tumor cells, therefore they kill tumor cells by intracellular conversion of prodrug 5-FC to cytostatic drug 5-FU and to 5-FUMP [7]. Similar observation we observe with tk*HSV*-MSC/GCV system. Tumor specificity of MSC suicide gene exosomes is rather broad, but tissue origin of MSCs can influence the tumor cell targeting and augment the suicide gene therapeutic effect. Exosomes secreted by MSCs derived from placenta selectively inhibit growth of prostate cancer cells. Generally, MSC-exosomes display a unique anti-tumor effect thanks to a variety of molecular species such as micro-RNAs within their cargo and the capability to activate multiple gene pathways [8]. Since MSCs have the remarkable tendency to home in tumors, their exosomes retain the homing property of parent cells. For instance dental pulp MSCs, being of neural crest-origin cells, migrate to intracerebral glioblastomas after intranasal administration [9].

Exosomes from suicide gene modified MSCs have the potential to be a new class of highly selective cancer-targeted therapeutics. Composition of exosomes released from MSCs might be purposely modified to create therapeutically interesting exosomes for cancer treatment. Recently we reported a dual action of MSCs-yCD-UPRT-MSCs exosomes possessing beside mRNA of suicide gene also iron oxide nanoparticles. These nanoparticles were able to act both as a prodrug dependent therapeutic and hyperthermia inducing factor when tumor cells were exposed to alternating magnetic field [10].

Our present research direction is headed to development of suicide gene exosomes for metastases. Human tumor cell specific exosomes with suicide gene we found to induce tumor cell death in a similar way as MSC exosomes (article in preparation). It was reported that aggressive tumor cells release exosomes creating pre-metastatic niche in body organs. Integrins of tumor exosomes determine organotropic metastasis. Therefore suicide gene exosomes prepared from primary human tumor might be used in future as a therapeutic tool for the patient-tailored therapy of metastases. The usefulness of prodrug gene therapy for cancer mediated by MSC suicide gene exosomes is now validated by animal studies.

Described findings open new exciting possibilities for cancer gene therapy of human tumors that do not respond to standard therapies such as grade IV glioblastoma multiform. MSC suicide gene exosomes carry mRNA of suicide gene protected by the plasma membrane. They can travel long distances in the body, when administered intravenously might execute their therapeutic effect at a distant location. Accumulating evidence indicates that cancer therapy using MSC exosomes has multiple advantages over cell therapy. CM or exosomes are stable after intravenous administration and exhibit a superior safety profile.

**Figure 1:**
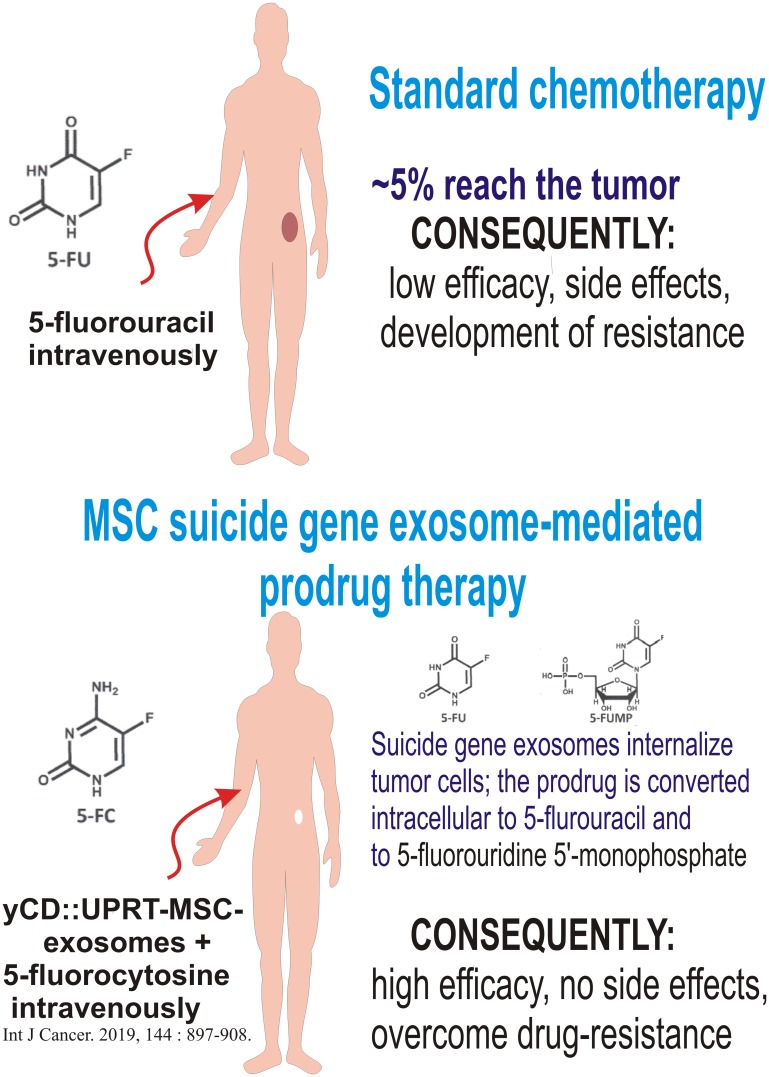
MSC-suicide gene exosomes as an intracellular cancer drug.

To be innovative cancer therapy curative; it must differ from standard therapies. The therapy should be targeted not only to tumor mass, but also specifically to tumor cells, drugs should be able to internalize both cancer stem cells and cancer cells and act intracellular. In this way it could be possible to avoid side effects, to prevent development of resistance and even kill already resistant tumor cells (Figure 1). Suicide gene exosomes fulfill these requirements.
